# Fish Meal Replacement by Chicken By-Product Meal in Diet: Impacts on Growth and Feed Availability of Juvenile Rockfish (*Sebastes schlegeli*), and Economical Analysis

**DOI:** 10.3390/ani15010080

**Published:** 2025-01-02

**Authors:** Ran Li, Sung Hwoan Cho

**Affiliations:** 1 Xingzhi College, Zhejiang Normal University, Jinhua 321004, China; ytliran@126.com; 2 Division of Convergence on Marine Science, Korea Maritime and Ocean University, Busan 49112, Republic of Korea

**Keywords:** rockfish (*Sebastes schlegeli*), fish meal replacement, chicken by-product, blood chemistry, economic profit index

## Abstract

**Simple Summary:**

Fish meal is one of the most expensive components in formulating fish feeds. Finding a substitute that is an inexpensive and supply-stable protein source for fish meal will lower the feed cost and achieve sustainable fish culture. Chicken by-products can be considered a viable and innovative option as a replacer for FM in aquafeeds because of their substantial protein and lipid content, year-round availability, and inexpensive price. This study revealed that fish meal up to 20% could be replaced with chicken by-product meal in rockfish feeds without impairing growth, feed availability, chemical composition, amino acid profiles, or blood parameters. This research will be helpful for feed producers to lower feed cost and provide a reference case for other aquafeed producers to utilize chicken by-products to formulate low-fish meal diets.

**Abstract:**

A 56-day feeding experiment was carried out to evaluate the effects of substituting fish meal (FM) with chicken by-product meal (CBM) in diets on the growth and feed utilization of rockfish (*Sebastes schlegeli*). Six experimental diets were formulated to be isonitrogenous and isolipidic. The control (Con) diet included 55% FM. In the Con diet, 10%, 20%, 30%, 40%, and 50% of FM was replaced with CBM, named as the CBM_10_, CBM_20_, CBM_30_, CBM_40_, and CBM_50_ diets, respectively. A total of 540 juvenile fish were distributed into 18 tanks (30 fish per tank and 3 tanks per diet) and fed to apparent satiation two times daily for 56 days. The weight gain and specific growth rate of rockfish fed the CBM_10_ and CBM_20_ diets were comparable to rockfish fed the Con diet. The feed consumption of rockfish fed the Con and CBM_10_ diets was significantly (*p <* 0.001) higher than that of fish fed all other diets, except for the CBM_20_ diet. However, protein retention, biometric indices, chemical composition, amino acid profiles, and plasma and serum parameters of rockfish were not significantly influenced by dietary FM substitution with CBM. The Con, CBM_10_, and CBM_20_ diets showed superior (*p <* 0.001) economic profit index (EPI) compared to the CBM_30_, CBM_40_, and CBM_50_ diets. Conclusively, FM up to 20% could be substituted by CBM in diets without impairing growth, feed availability, chemical composition, amino acid profiles, and blood parameters of rockfish grown from 2.5 g to 12.5 g. However, the long-term effects of CBM substitution or the potential use of combined CBM and other alternative protein sources for FM in rockfish diets are needed in future.

## 1. Introduction

Protein sources are the most expensive and crucial components in aquafeeds [1]. Fish meal (FM) is generally applied as the primary source of protein in most formulated carnivorous fish diets, not only because of its substantially balanced amino acid (AA) content, excellent palatability and digestibility, and absence of anti-nutritional factors (ANFs), but also because of its abundance of nucleotides and water- and fat-soluble vitamins [2,3]. The demand for FM is steadily growing because of the swift expansion of global aquaculture and the aquafeed industry. The overfishing of wild fish stocks, however, has decreased its availability and raised its market price over the past few decades [4]. Thus, finding an inexpensive, supply-stable, and environmentally friend alternative protein source for FM will help to lower the feed cost, relieve the pressure on FM supply, and achieve sustainable fish culture.

Numerous FM replacers in fish feeds have been explored during the last few decades. Unlike plant-origin protein sources, in general, animal-origin protein sources have excellent characteristics, in terms of high content of protein, balanced AA, digestible minerals, and a lack of ANFs [5,6]. As a substitute to FM, various animal protein sources in aquafeeds, such as meat meal (MM) [7], poultry by-product meal (PBM) [8,9], insect meal [10], tuna by-product meal (TBM) [11,12], meat and bone meal (MBM) [8,9], hydrolyzed feather meal [13], blood meal [14], and chicken plasma powder [15], have been evaluated. Still, many scientists are looking for a FM replacer that can used as an inexpensive and supply-stable protein source in fish feeds. Chicken by-product meal (CBM) has attracted the interest of feed nutritionists because of its substantial protein and lipid content, year-round availability, and inexpensive price compared with other animal protein sources.

In 2021, the global production of chicken meat was more than 73 million metric tons (MT), whereas it was recorded to be 935,000 MT in the Republic of Korea (*hereafter*, Korea) [16]. In chicken processing plants, several thousand MTs of chicken by-products are continuously produced, such as heads, bones, feet, blood, feathers, and viscera [17]. Chicken by-products are exclusively produced at chicken processing plants, whereas poultry by-products are produced by all domestic bird processing plants, such as those for ducks and geese. CBM is made of dry ground rendered clean portions of chicken carcasses [18] and can be considered a viable and innovative option for FM replacement in aquafeeds. In Ha et al.’s [17] study, 50% FM could be replaced with CBM in the olive flounder (*Paralichthys olivaceus*) diet without bringing about unfavorable impacts on growth in a 56-day feeding trial. Additionally, 10% FM protein in a 35% FM-basal diet was replaced with chicken waste meal without causing adverse effects on weight gain and feed availability in Asian seabass (*Lates calcarifer*) [19].

Rockfish (*Sebastes schlegeli*) is a widely farmed marine fish species in Korea, China, and Japan [20]. Numerous benefits, including rapid growth, excellent endurance of low water temperatures, and easy seeding production, have made rockfish one of Korea’s most representative farmed fish species [21,22]. As the second most popular marine fish species after olive flounder, the aquaculture production of rockfish was reported to be 14,429 MT in Korea in 2023 [23]. FM substitutions with other proteins, such as dehulled or fermented soybean meal (SBM) [24,25] and TBM [11], in rockfish diets have been demonstrated previously. Recently, Lee et al. [7] stressed that FM up to 80% could be replaced with MM in the diet of rockfish without deteriorating growth and feed utilization when juvenile fish were fed with a 55% FM-based diet, or one of diets with 10%, 20%, 40%, 60%, 80%, and 100% replacements of FM with MM. In particular, Yan et al. [9] proved that the substitution of 15% FM with PBM, MBM, SBM, and cottonseed meal could be applied in the diets of rockfish without retarding specific growth rate (SGR) when juvenile fish were provided with a 72.5% FM-basal diet or a diet substituting 15% FM with PBM, MBM, SBM, cottonseed meal or canola meal for 56 days. Furthermore, the greatest SGR was found in rockfish fed a diet replacing 15% FM with PBM, but this study only evaluated the substitutability of the fixed (15%) level of FM with various protein sources in the rockfish diet containing unusually high (72.5%) FM, and did not assess the substitutability of different levels of FM with respective substitutes in rockfish diets containing practical levels of FM.

This experiment, therefore, was performed to elucidate the effects of replacing various levels of FM with CBM in rockfish diets on the growth, feed availability, blood chemistry, biochemical composition, and economical factors.

## 2. Materials and Methods

### 2.1. Preparation of the Experimental Diets

A commercially available CBM was obtained from Chamfre Co. Ltd. (Buan-gun, Jeollabuk-do, Republic of Korea). Here, 55% FM (blended sardine meal:anchovy meal = 1:1) and 17.5% fermented SBM were employed as the main sources of protein in the control (Con) diet (Table 1). Additionally, 19% wheat flour and 3% each of fish and soybean oils were included in the Con diet as the carbohydrate and lipid sources, respectively. In the Con diet, 10%, 20%, 30%, 40%, and 50% of FM were substituted with CBM, referred to as the CBM_10_, CBM_20_, CBM_30_, CBM_40_, and CBM_50_ diets, respectively. By adjusting the content of wheat flour and soybean oil, six experimental diets were formulated to be isonitrogenous at 51.0% and isolipidic at 12.5% to ensure appropriate protein and lipid contents for the growth of rockfish [7]. All experimental diets were assigned to triplicate groups of fish.

The ingredients of the experimental diets were thoroughly mixed with water (3:1) and then extruded into pellets and dried at 40 °C for 24 h. The experimental diets were stored at −20 °C until use.

### 2.2. Preparation of Rockfish and Feeding Conditions

Juvenile fish of similar sizes were purchased from a hatchery (Buan-gun, Jeollabuk-do, Republic of Korea). To acclimate to the culturing conditions, fish were stocked in a 5 ton tank for 2 weeks and provided with commercial feed (55% crude protein and 8% crude lipid; National Federation of Fisheries Cooperatives Feed, Uiryeong-gun, Gyeongsangnam-do, Republic of Korea). Upon completion of the 2-week acclimatization period, a total of 540 rockfish (initial weight of 2.4 ± 0.01 g; mean ± SEM) were randomly distributed into 18 50 L flow-through tanks (water volume 40 L, 30 juvenile/tank). Throughout the experimental period, 4.3 L/min of mixed underground seawater and sand-filtered seawater (1:1) was supplied to each tank. The proper aeration was continuously provided to each tank, and the photoperiod was left natural. Rockfish were fed to apparent satiation two times daily (08:30 and 17:30) for 56 days. Throughout the 56-day feeding trial, the water temperature changed from 16.1 to 21.8 °C, the salinity changed from 30.8 to 32.4 g/L, the dissolved oxygen changed from 7.3 to 8.2 mg/L, and the pH changed from 7.4 to 7.8. All experimental tanks were siphon-cleaned daily.

### 2.3. Measurements of Growth and Biological Indices of Rockfish

After the completion of the 56-day feeding trial, all live rockfish from each tank were anesthetized with tricaine methanesulfonate at 100 mg/L, based on the approach of Son et al.’s [26] study, and then counted and weighed collectively after the 24 h fasting. The total final weight and total number of fish from each tank were measured to evaluate the weight gain, SGR, and survival of fish. Eight fish were individually measured for their total length and weight to calculate the condition factor (*K*). After that, these rockfish were dissected to separate the visceral and liver organs and thus evaluate the viscerosomatic index (VSI) and hepatosomatic index (HSI), respectively.

### 2.4. Biochemical Composition Analysis of Experimental Diets and Fish

Ten juvenile fish prior to the experiment and 7 fish from each tank after the 56-day feeding trial were randomly chosen for analyses of the biochemical composition of the whole body of the rockfish. The crude protein and crude lipid contents of the experimental diets and fish were analyzed using the Kjeldahl (KjeltecTM 2100 Distillation Unit; Foss, Hillerød, Denmark) and ether extraction (SoxtecTM 2043 Fat Extraction System; Foss, Hillerød, Denmark) methods, respectively. The moisture contents of the experimental diets and fish were dried in a dry oven at 105 °C for 4 and 24 h, respectively. The ash contents of the samples were determined using a muffle furnace at 550 °C for 4 h.

In addition to methionine, cysteine, and tryptophan, all AA from the experimental diets and whole-body fish were analyzed using an AA analyzer (L-8800 Auto-analyzer; Hitachi, Ltd., Tokyo, Japan) followed by ion-exchange chromatography after hydrolysis with 6 N HCl at 110 °C for 24 h. To analyze the methionine and cysteine in diets, the samples were oxidized with performic acid at below 5 °C for a day to attain methionine sulfone and cysteic acid, and then lyophilized twice with deionized water, hydrolyzed, and assayed in accordance with the AA standard analytical procedure. Tryptophan was hydrolyzed by NaOH (4.2 mol/L) at 110 °C for 24 h and then measured via high-performance liquid chromatography (S1125 HPLC pump system; Sykam GmbH, Eresing, Germany).

The fatty acids (FA) of the samples were analyzed by comparison with the standard samples (37-component FAME mix CRM47885; Supelco™, St. Louis, MO, USA). Lipids were obtained for the FA analysis of the samples with a blend of chloroform and methanol (2:1 *v*/*v*), based on the method of Folch et al.’s [27] study, and FA methyl esters were prepared by transesterification with 14% BF_3_-MeOH (Sigma, St. Louis, MO, USA).

### 2.5. Plasma and Serum Parameters of Fish

Blood was collected from the caudal veins of ten fish using five heparinized syringes and five non-heparinized syringes. Plasma and serum samples were separated after centrifugation (2700× *g*) at 4 °C for 10 min, and then stored at −70 °C until use. Plasma was utilized for the detection of alanine aminotransferase (ALT), aspartate aminotransferase (AST), total bilirubin (TB), alkaline phosphatase (ALP), total cholesterol (T-CHO), triglyceride (TG), total protein (TP), and albumin (ALB), with an automatic analyzer (Fuji Dri-Chem NX500i, Fujifilm, Tokyo, Japan). Serum was utilized for the evaluation of superoxide dismutase (SOD) and lysozyme activity, as reported in our previous research [28].

### 2.6. Economic Parameters of the Study

The economic return of the study was evaluated by applying the formula previously reported by Martínez-Llorens et al. [29]—economic conversion ratio (ECR, USD/kg) = feed consumption (kg/fish) × diet price (USD/kg)/weight gain of fish (kg/fish), economic profit index (EPI, USD/fish) = final weight (kg/fish) × fish sale price (USD/kg)—feed consumption (kg/fish) × diet price (USD/kg). The prices (USD/kg, 1 USD = KRW 1304) of these ingredients were as follows: FM = 2.11, CBM = 0.88, fermented SBM = 0.66, wheat flower = 0.52, fish oil = 2.61, soybean oil = 1.69, vitamin mix = 7.82, mineral mix = 6.29, and choline chloride = 1.23. The price of juvenile rockfish was calculated at USD 1.00/10 g juvenile fish.

### 2.7. Statistical Analysis

One-way ANOVA was conducted in SPSS (SPSS Inc., Chicago, IL, USA) to analyze significant differences (*p <* 0.05) between dietary treatments. Tukey’s test was applied for post-hoc analysis when a significant difference was noticed among dietary treatments. All percentage data were arcsine-transformed before statistical analysis. The orthogonal polynomial contrasts were used to assess the response for all dependent variables (linear, quadratic, or cubic), and regression analysis was performed to identify the most suitable model with different replacement s of FM with CBM and different dependent variables.

## 3. Results

### 3.1. AA and FA Profiles of the Experimental Diets

The protein sources of FM and CBM had similar AA profiles, except for the fact that FM had a relatively higher lysine and aspartic acid content than CBM, while CBM had relatively higher arginine, glycine, and proline contents than FM (Table 2). The AA profiles of the experimental diets, except for glycine and proline, were well reflected from those of FM and CBM. The contents of arginine, phenylalanine, tryptophan, and valine among the essential AAs (EAAs) in the experimental diets increased with elevated levels of FM replacement with CBM. Leucine and glutamic acid were found to be the most abundant EAAs and NEAAs in the experimental diets, respectively.

The eicosapentanoic acid (EPA, 20:5n-3), docosahexanoic acid (DHA, 22:6n-3), arachidonic acid (ARA, 20:4n-6), and total n-3 highly unsaturated FA (∑n-3 HUFA) contents in CBM were lower than those in FM (Table 3). However, the contents of total saturated fatty acids (∑SFA) and monounsaturated FA (∑MUFA) in CBM were higher than those in FM. The levels of ∑SFA and ∑MUFA in the experimental diets increased with elevated levels of FM substitution with CBM, but the ∑n-3 HUFA and ARA levels decreased.

### 3.2. Growth of Rockfish

**Figure 1:**
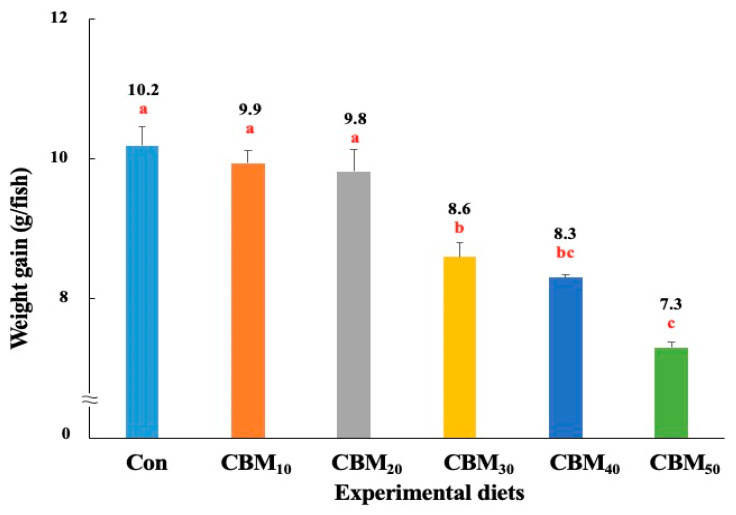
Weight gain (g/fish) of rockfish (*Sebastes schlegeli*) fed the experimental diets for 56 days (means of triplicate ± SE) (*p <* 0.001). The orthogonal polynomial contrast (linear, *p =* 0.001; quadratic, *p =* 0.035; cubic, *p =* 0.399) and the best-fitting model show a linear (Y = –0.0583X + 10.4905, *p <* 0.001, adjusted R^2^ = 0.8494) relationship between the weight gain of rockfish and dietary replacement levels of fish meal (FM) with chicken by-product meal (CBM). The different letters under numerical values indicate significant differences among dietary treatments.

**Figure 2:**
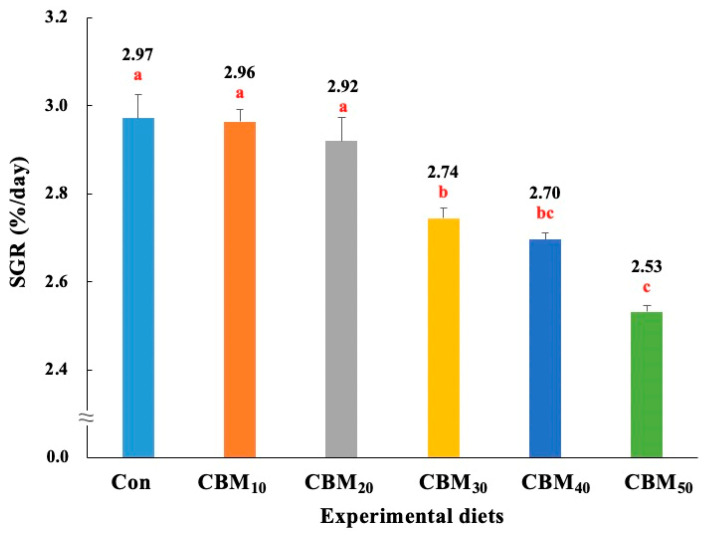
Specific growth rate (SGR, %/day) of rockfish (*Sebastes schlegeli*) fed the experimental diets for 56 days (means of triplicate ± SE) (*p <* 0.001). The orthogonal polynomial contrast (linear, *p =* 0.001; quadratic, *p =* 0.032; cubic, *p =* 0.441) and the best-fitting model show a linear (Y = –0.0091X + 3.0319, *p <* 0.001, adjusted R^2^ = 0.8329) relationship between the SGR of rockfish and dietary replacement levels of fish meal (FM) with chicken by-product meal (CBM). SGR (%/day) = [(Ln final weight of rockfish − Ln initial weight of rockfish) × 100]/days of feeding trial. The different letters under numerical values indicate significant differences among dietary treatments.

The survival of rockfish varied from 94.4% to 97.8%, and it was not significantly (*p* > 0.7) affected by dietary FM replacement with CBM (Table 4). However, rockfish fed the Con, CBM_10_, and CBM_20_ diets showed significantly (*p <* 0.001) greater levels of weight gain (Figure 1) and SGR (Figure 2) than fish fed the CBM_30_, CBM_40_, and CBM_50_ diets. Rockfish fed the CBM_50_ diet showed the poorest weight gain and SGR. Based on orthogonal polynomial contrast, the weight gain and SGR of rockfish exhibited significant linear (*p =* 0.001 for both) and quadratic (*p =* 0.035 and *p =* 0.032, respectively) relationships with dietary FM substitution by CBM. The most appropriate models between dietary FM substitution by CBM and weight gain (Y = −0.0583X + 10.4905, *p <* 0.001, adjusted R^2^ = 0.8494) and SGR (Y = –0.0091X + 3.0319, *p <* 0.001, adjusted R^2^ = 0.8329) were observed.

### 3.3. Feed Availability and Bioligical Indices of Fish

**Figure 3:**
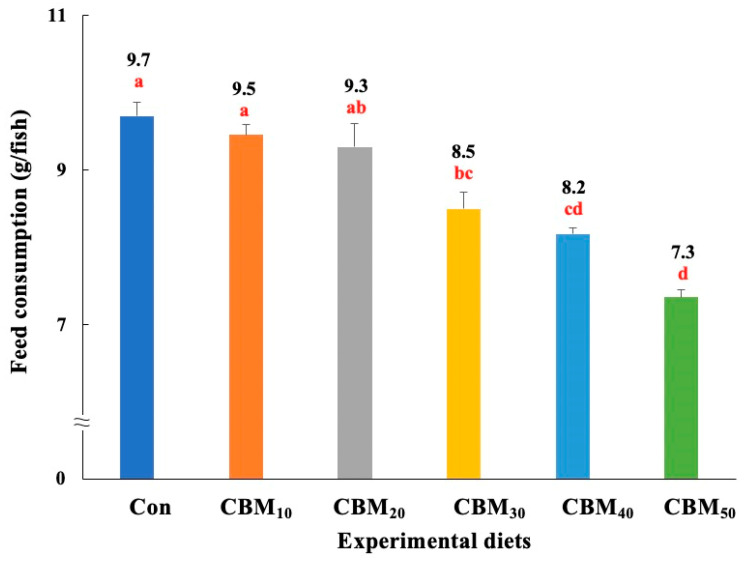
The feed consumption (g/fish) of rockfish (*Sebastes schlegeli*) fed the experimental diets for 56 days (means of triplicate ± SE) (*p <* 0.001). The orthogonal polynomial contrast (linear, *p =* 0.001; quadratic, *p =* 0.058; cubic, *p =* 0.851) and the best-fitting model show a linear (Y = −0.0468X + 9.9173, *p <* 0.001, adjusted R^2^ = 0.8508) relationship between the feed consumption of rockfish and dietary replacement levels of fish meal (FM) with chicken by-product meal (CBM). The different letters under numerical values indicate significant differences among dietary treatments.

The feed consumption of rockfish fed the Con and CBM_10_ diets were significantly (*p <* 0.001) higher than those of rockfish fed the CBM_30_, CBM_40_, and CBM_50_ diets, but not significantly (*p* > 0.05) different from that of rockfish fed the CBM_20_ diet (Figure 3). With respect to the orthogonal polynomial contrast, the feed consumption of fish exhibited a significant linear (*p =* 0.001) relationship with FM substitution by CBM in diets. The most fitting model between dietary substitution of CBM for FM and feed consumption (Y = –0.0468X + 9.9173, *p <* 0.001, adjusted R^2^ = 0.8508) was observed. Rockfish fed the Con, CBM_10_, and CBM_20_ diets achieved significantly (*p <* 0.001) higher FER than rockfish fed the CBM_40_ and CBM_50_ diets, but these values were not significantly (*p* > 0.05) different from those of rockfish fed the CBM_30_ diet. The PER of rockfish fed the CBM_20_ diet was significantly (*p* < 0.002) greater than that of fish fed the CBM_40_ and CBM_50_ diets, but was not significantly (*p* > 0.05) different from that of rockfish fed the Con, CBM_10_, and CBM_30_ diets. Based on the orthogonal polynomial contrast, the FER and PER of rockfish exhibited significant linear (*p =* 0.001) and quadratic (*p =* 0.025) relationships, and a linear (*p =* 0.001) relationship, respectively, with FM replacement with CBM in diets. The most fitting models between dietary replacement of CBM for FM and FER (Linear, *p <* 0.001, adjusted R^2^ = 0.657) and PER (Linear, *p <* 0.001, adjusted R^2^ = 0.617) were observed. PR, as well as *K*, HSI, and VSI, were not significantly (*p* > 0.05 for all) influenced by dietary FM replacement with CBM.

### 3.4. Biochemical Composition of the Whole-Body Fish

The moisture, crude protein, crude lipid, and ash contents of the whole-body rockfish varied from 72.0 to 73.2%, 15.2 to 15.6%, 6.8 to 7.3%, and 4.0 to 4.3%, respectively, and these parameters were not significantly (*p* > 0.05 for all) influenced by dietary FM substitution by CBM (Table 5).

Dietary FM substitution with CBM had no significant impact on the AA profiles of the whole bodies of rockfish (Table 6).

All FA profiles of the whole bodies of rockfish, except for C22:0, C24:0, and C22:1n-9, were significantly (*p <* 0.05) affected by dietary FM replacement with CBM (Table 7). The FA profiles of the whole bodies of fish are well reflected in the dietary FA profiles. Specifically, ∑MUFA increased, but EPA, DHA, and ∑n-3 HUFA decreased, with greater dietary FM substitution by CBM. Based on the orthogonal polynomial contrast, the ∑SFA of rockfish exhibited a remarkable linear (*p =* 0.001) relationship, ∑MUFA exhibited significant linear (*p =* 0.001), quadratic (*p =* 0.001), and cubic (*p =* 0.001) relationships, and the ∑n-3 HUFA showed significant linear (*p =* 0.001) and quadratic (*p =* 0.025) relationships with elevated dietary FM substitution with CBM. The most suitable models between dietary replacement of CBM for FM and ∑SFA (Linear, *p <* 0.001, adjusted R^2^ = 0.498), ∑MUFA (Linear, *p <* 0.001, adjusted R^2^ = 0.979), and ∑n-3 HUFA (Linear, *p <* 0.001, adjusted R^2^ = 0.943) were noted.

### 3.5. Plasma and Serum Parameters of Fish

The plasma AST changed from 147.7 to 151.3 U/L, ALT changed from 22.3 to 25.3 U/L, ALP changed from 184.3 to 189.7 U/L, TB changed from 1.2 to 1.8 mg/dL, T-CHO changed from 252.7 to 256.0 mg/dL, TG changed from 360.7 to 364.7 mg/dL, TP changed from 4.2 to 4.7 g/dL, and ALB changed from 1.0 to 1.5 g/dL. All these parameters were not significantly (*p* > 0.05 for all) influenced by the replacement of dietary FM with CBM (Table 8).

The serum SOD changed from 2.4 to 2.7 ng/mL and lysozyme activity changed from 304.3 to 485.2 U/mL. No significant (*p* > 0.8 and *p* > 0.4, respectively) differences in these parameters were noticed.

### 3.6. Economic Analysis of the Study

The prices of the experimental diets tended to decrease with increased levels of FM replacement with CBM (Table 9). The ECR values of the CBM_40_ and CBM_50_ diets were significantly (*p <* 0.001) lower than those of the Con, CBM_10_, CBM_20_, and CBM_30_ diets. However, the EPI of the Con, CBM_10_, and CBM_20_ diets were significantly (*p <* 0.001) higher than those of the CBM_30_, CBM_40_, and CBM_50_ diets. The ECR and EPI values of the experimental diets exhibited significant linear (*p =* 0.001 for both) and quadratic (*p =* 0.035 and *p =* 0.001, respectively) relationships with dietary FM substitution with CBM. The most fitting models between FM substitution with CBM in diets and ECR (Linear, *p <* 0.001, adjusted R^2^ = 0.878) and EPI (Linear, *p <* 0.001, adjusted R^2^ = 0.852) were observed.

## 4. Discussion

The lack of significant differences in the weight gain, SGR, or feed consumption of rockfish fed the Con, CBM_10_, and CBM_20_ diets in this study indicates that FM up to 20% could be substitutable by CBM without compromising weight gain, SGR, or feed consumption. However, Ha et al. [17] emphasized that 50% FM could be substituted with CBM in the 65% FM-based diet of olive flounder without causing any undesirable impacts on growth or feed availability. In considering the substitutability of the same CBM for FM up to 20% in the rockfish diet in this study and 50% in the olive flounder diet in Ha et al.’s [17] study, we see that its substitutability for FM seems to vary depending on fish species. The replacement of FM protein with chicken waste meal up to 10% could be performed in the diets of Asian seabass without deteriorating weight gain and feed availability when juvenile fish were supplied with a 35% FM-based diet, or one of diets replacing 5%, 10%, 15%, or 20% FM protein with chicken waste meal [19].

Nutritional quality should be considered before incorporating a new feed ingredient into aquafeeds [33]. The crude protein content (69.7%) in FM is relatively high compared to that (65.4%) in CBM, while the crude lipid content (17.6%) in the latter is relatively high compared to that (11.1%) in the former, as described in this study. Another critical element influencing the utilization of an alternative protein for FM in aquafeeds is the abundancy or balance of EAA [34]. The relatively high content of EAA in FM, especially in lysine and methionine, compared to CBM should be considered in this study because dietary deficiencies in EAA led to poor growth in fish [35]. Although increased FM substitution with CBM lowered the lysine content in the experimental diets, the lysine content (3.07–3.13% of the diet) in all experimental diets met the requirement in rockfish (2.99% of the diet) [30]. Additionally, it has been proven that the dietary inclusion of cysteine could spare approximately 40–50% of the methionine requirements in yellowtail kingfish (*Seriola lalandi*) and red drum (*Sciaenops ocellatus*), respectively [36,37]. Although methionine content ranged from 0.98% in the CBM_50_ diet to 1.13% in the Con diet, in this study, the dietary methionine content was lower than the requirement (1.37% of the diet) in rockfish reported by Yan et al. [31], while the cysteine contents reported here (0.69–0.74% of the diet) in relation to all experimental diets are higher than the cysteine contents (0.12% of the diet) reported in their study. Thus, the relatively low methionine contents seen in all experimental diets in this study might not cause any detrimental effects on the growth of fish.

In order to achieve appropriate growth, marine finfish need sufficient n-3 HUFA, especially EPA and DHA [38,39]. Except for in the CBM_50_ diet (6.60% of total FA), the ∑n-3 HUFA (7.64–11.92% of total FA) in all experimental diets met its requirement (7.20% of total FA) for rockfish [32]. Thus, the lowest ∑n-3 HUFA, seen in the CBM_50_ diet, might be a main factor contributing to the observation of the poorest growth of rockfish in this experiment. Apart from ∑n-3 HUFA, ARA is also crucial for the growth of Japanese seabass (*Lateolabrax japonicas*) [39] and javelin goby (*Synechogobius hasta*) [40]. Because ARA tended to decrease with the elevated substitution of dietary FM with CBM in this experiment, this might be another reason for the observation of the poorer growth of rockfish fed the higher FM-replaced diets. However, to our knowledge, none of the specific dietary EFA requirements for rockfish are yet known.

The weight gain and SGR of rockfish appeared to be associated with feed consumption in this experiment. The weight gain of rockfish directly resulted from the linear reduction in feed consumption with elevated levels of dietary FM replacement with CBM, according to the results of the regression analysis and orthogonal polynomial contrast test. Feed consumption shown by rockfish were significantly decreased, especially when the level of dietary CBM replacement with FM was higher than 20%. The reduced palatability of low-FM diets with more than 20% FM replaced with CBM might be the main reason for the reduced feed consumption and weight gain of rockfish. Improvements in feed intake by fish could be explained by the excellent palatability of the protein source included, or the leaching of smell-releasing chemical compounds from the protein source into the water, which stimulated the feed-searching behavior of fish [41]. Dietary histidine content was closely associated with growth performance (weight gain and SGR) and feed consumption in rockfish [42]. Since the histidine content in the experimental diets decreased with elevated FM substitution with CBM in this study, this could be another reason why high (30–50%) dietary FM substitution with CBM led to reduced feed intake in rockfish. Similarly, increased levels of FM substitution with PBM in diets deteriorated the palatability of low-FM diets and feed intake, and eventually reduced the weight gain of fish [2,43]. Lee et al. [24] and Wang et al. [44] also emphasized that the reduced palatability of low-FM diets might be responsible for the decreased feed intake and growth of fish. The incorporation of feed enhancer or attractant into low-FM feeds may be an excellent strategy to enhance the palatability of low-FM diets and increase feed consumption by fish. Furthermore, the FER and PER of rockfish were significantly decreased compared to those of rockfish fed the Con diet in this experiment, especially when the levels of dietary CBM substitution for FM were higher than 40 and 50%, respectively. Likewise, the substitution of dietary FM with alternative proteins lowered the FER and/or PER of rockfish [25], olive flounder [12], and Japanese seabass [33]. However, Ha et al. [17] found that the FER and PER of olive flounder were not significantly affected by increased FM substitution with CBM in diets.

The biological indices of fish, such as *K* and HSI, could reveal information on growth, health conditions, nutritional status, and tolerance to environmental challenges [45,46]. Sabarudin et al. [47] stressed that heavier fish within a specific length (higher *K*) are in a better condition. However, none of the values of K, HSI, or VSI of rockfish were significantly altered by increased FM substitution with CBM in diets in this experiment. Likewise, elevated dietary FM replacement with TBM [11], MM [7], and fermented SBM [24] did not change the *K*, VSI, or HSI of rockfish. However, elevated dietary FM substitution by PBM significantly altered the HSI of black sea bream (*Acanthoparus schlegelii*), but not the *K* and VSI [45].

The chemical compositions and AA profiles of the whole bodies of rockfish were unaffected by elevated FM substitution with CBM in diets in this experiment. This might indicate that the substitution of dietary FM with up to 50% CBM did not cause any negative impacts on the chemical composition or AA profiles of the whole bodies of rockfish. Similarly, elevated dietary FM substitution with CBM did not change the chemical compositions or AA profiles of olive flounder [17], or the chemical composition of abalone carcass (*Haliotis discus hannai*) [48], also implying that CBM has no undesirable impact on the chemical compositions of aquatic animals. However, the crude protein and ash contents of the whole bodies of seabass decreased, but the crude lipid content increased with elevated dietary FM replacement with chicken waste meal [19]. Lee et al. [24] suggested that the replacement of 24% FM protein with fermented SBM did not influence the proximate AA profiles of the whole bodies of rockfish in an 8-week feeding trial. However, there were also contradicting results showing that the chemical compositions and/or AA profiles of the whole bodies of fish were significantly impacted by the replacement of dietary FM with other proteins [5,11,45,49].

Elevated dietary FM substitution with CBM led to increased ∑MUFA in the whole bodies of rockfish, but decreased DHA, EPA, and ∑n-3 HUFA, in this study. DHA and EPA are critical for the development and maintenance of the health of marine fish [32] or carnivorous fish species [50]. However, in this study, EPA and DHA in the whole bodies of rockfish were significantly decreased with elevated levels of FM replacement with CBM in diets. Furthermore, the FA profiles of the whole bodies of rockfish closely matched the dietary FA profiles in this experiment, agreeing with the results of previous reports [13,51], in which the FA profiles of fish were closely associated with the dietary FA profiles.

Plasma parameters, trustworthy markers of fish’s health and physiological condition, are very important in fish farming [52]. Among the blood parameters, AST and ALT are commonly employed to assess the condition of the liver since they are closely related to fish liver cell damage [53]. Additionally, elevated values of AST and ALT in plasma are considered indicators of liver damage [54]. Kader et al. [55] revealed that replacing 60% of FM in the diet with soybean protein concentrate alone increased the plasma AST and ALT in red sea bream (*Pagrus major*). However, no significant impacts of dietary FM substitution with CBM on plasma measurements in rockfish were found in this experiment, agreeing with Jeon et al. [11] and Jeong et al.’s [56] studies, in which the plasma chemistry of rockfish was not significantly influenced by FM substitution with TBM and mealworm meal (*Tenebrio molitor*) in diets. Likewise, the substitution of FM with animal proteins in diets had no impact on the plasma chemistry of rockfish [7,57], olive flounder [17], or black sea bream [45].

SOD is the most critical antioxidant enzyme combating free radicals in live organisms [58]. Lysozyme plays a mediating role against microbial invasion, making it an essential defense molecule [59]. Both SOD and lysozyme activity are well-known parameters in fish that are commonly used as the markers of stress response and disease resistance [60]. In this study, elevated dietary FM substitution with CBM had no undesirable impacts on SOD or lysozyme activity in rockfish. Likewise, the replacement of dietary FM with CBM did not influence SOD or lysozyme activity in olive flounder [17]. Several studies also proved that the substitution of various alternative protein sources for FM in diets led to no significant change in SOD or lysozyme activity in rockfish [7], or lysozyme activity in Atlantic salmon (*Salmo salar*) [61] and European seabass (*Dicentrarchus labrax*) [13]. In contrast to the findings of this experiment, the replacement of dietary FM with PBM and SBM significantly changed the lysozyme activity and/or SOD of fish [62,63]. Therefore, the stress responses of fish may differ depending on fish species, alternative protein sources to FM, and the FM substitution level in the diet.

The price and ECR of the experimental diets tended to decrease with elevated levels of FM substitution with CBM in this study. Nevertheless, the higher EPI of the Con, CBM_10_, and CBM_20_ diets compared to the CBM_30_, CBM_40_, and CBM_50_ diets demonstrates that rockfish farmers could achieve higher economic returns by adopting the former diets compared to the latter diets. This result is also consistent with the outputs of the weight gain, SGR, and feed consumption of rockfish based on multiple comparison tests performed in the present study. Considering the unstable production and increasing price of FM [4], and the globally stable supply of CBM from chicken meat processing plants [16], the cost advantage of CBM is apparent, and the utilization of CBM in fish feeds will create high benefits for fish farmers.

The results of this study are basically consistent with the results of Yan et al.’s [9] research, in which 15% of FM could be replaced with PBM in diets without deteriorating the growth or FE of rockfish. Similarly, 25% of FM could be replaced with PBM in the 77.3% FM-based diets of black sea turbot (*Psetta maeotica*) without deteriorating growth, nutrient utilization, or nitrogen retention [64]. Nevertheless, the effects of various levels of FM replacement with CBM in rockfish feed on whole-body AA and FA profiles, blood parameters, and economic parameters are strongly comparable in the present study. This could help elucidate the effects of the replacement of dietary FM with CBM on the health and nutrition status of rockfish. However, the potential variability in the nutritional composition of CBM also depends on the source and processing methods. These factors could affect the universality of the application of the results of the current study. In addition, in the context of utilizing CBM in aquafeeds, long-term studies are needed to evaluate and address the potential environmental problems and fish health-related issues, such as the potential risks of disease transmission, the impacts on liver health, and the integrity of different organs [2,62,65].

## 5. Conclusions

FM could be substituted with CBM up to 20% in a 55% FM-based diet without deteriorating weight gain, SGR, or feed consumption in rockfish when growing from 2.5 g to 12.5 g. In addition, the replacement of FM with CBM in diets did not lead to any significant changes in the chemical composition, AA profiles, or blood parameters of rockfish. The long-term effects of CBM substitution, or the potential use of CBM combined with other alternative protein sources to FM, in rockfish diets are needed in the future.

## Figures and Tables

**Table 1:** Ingredient and chemical compositions of the experimental diets (%, dry matter basis).

	Experimental Diets					
	Con	CBM_10_	CBM_20_	CBM_30_	CBM_40_	CBM_50_
Ingredient (%, DM)						
Fish meal (FM) ^1^	55.0	49.5	44.0	38.5	33.0	27.5
Chicken by-product meal (CBM) ^2^		5.8	11.6	17.4	23.2	29.0
Fermented soybean meal ^3^	17.5	17.5	17.5	17.5	17.5	17.5
Wheat flour	19.0	19.1	19.2	19.3	19.4	19.5
Fish oil	3.0	3.0	3.0	3.0	3.0	3.0
Soybean oil	3.0	2.6	2.2	1.8	1.4	1.0
Mineral premix ^4^	1.0	1.0	1.0	1.0	1.0	1.0
Vitamin premix ^5^	1.0	1.0	1.0	1.0	1.0	1.0
Choline chloride	0.5	0.5	0.5	0.5	0.5	0.5
Nutrients (%, DM)						
Dry matter	98.4	98.8	98.4	98.3	97.5	98.7
Crude protein	50.9	51.1	50.9	50.6	50.9	50.7
Crude lipid	12.7	12.5	12.6	12.3	12.5	12.6
Ash	11.4	11.2	10.7	10.4	10.3	9.3

^1^ Fish meal (FM) (crude protein, 69.7%; crude lipid, 11.1%; ash, 16.7%) (blend of sardine meal and anchovy meal at the ratio of 1:1) imported from Peru was purchased from Daekyung Oil & Transportation Co., Ltd. (Busan city, Republic of Korea) (USD 2.11/kg FM, USD 1 = KRW 1304). ^2^ Chicken by-product meal (CBM) (crude protein, 65.4%; crude lipid, 17.6%; ash, 8.5%) was purchased from Chamfre Co., Ltd. (Buan-gun, Jeollabuk-do, Republic of Korea) (USD 0.88/kg). ^3^ Fermented soybean meal (crude protein, 53.2%; crude lipid, 1.6%; ash, 7.3%) (USD 0.66/kg). ^4^ Mineral premix contained the following ingredients (g/kg mix): MgSO_4_·7H_2_O, 80.0; NaH_2_PO_4_·2H_2_O, 370.0; KCl, 130.0; ferric citrate, 40.0; ZnSO_4_·7H_2_O, 20.0; Ca-lactate, 356.5; CuCl, 0.2; AlCl_3_·6H_2_O, 0.15; KI, 0.15; Na_2_Se_2_O_3_, 0.01; MnSO_4_·H_2_O, 2.0; CoCl_2_·6H_2_O, 1.0. ^5^ Vitamin premix contained the following amounts, which were diluted in cellulose (g/kg mix): L-ascorbic acid, 121.2; dl-α-tocopheryl acetate, 18.8; thiamin hydrochloride, 2.7; riboflavin, 9.1; pyridoxine hydrochloride, 1.8; niacin, 36.4; Ca-d-pantothenate, 12.7; myo-inositol, 181.8; D-biotin, 0.27; folic acid, 0.68; p-aminobenzoic acid, 18.2; menadione, 1.8; retinyl acetate, 0.73; cholecalciferol, 0.003; cyanocobalamin, 0.003.

**Table 2:** Amino acid (AA) profiles (% of the diet) of the main ingredients (FM and CBM) and experimental diets with the replacement of various levels of fish meal with chicken by-product meal.

	Ingredients		Requirement	Experimental Diets					
	FM	CBM		Con	CBM_10_	CBM_20_	CBM_30_	CBM_40_	CBM_50_
Essential amino acid (%)									
Arginine	3.51	3.96		2.61	2.70	2.77	2.81	2.82	2.87
Histidine	1.50	1.34		1.03	1.02	1.02	1.00	0.99	0.99
Isoleucine	2.47	2.43		1.70	1.70	1.69	1.69	1.68	1.66
Leucine	4.50	4.47		3.59	3.51	3.44	3.38	3.38	3.33
Lysine	4.82	4.20	2.99 ^1^	3.13	3.12	3.11	3.10	3.09	3.07
Methionine	1.75	1.29	1.37 ^2^	1.13	1.10	1.07	1.05	1.00	0.98
Phenylalanine	2.42	2.46		1.97	1.99	1.99	2.00	2.01	2.03
Threonine	2.69	2.53		2.00	2.00	1.99	1.99	1.98	1.97
Tryptophan	0.49	0.56		0.33	0.35	0.38	0.39	0.40	0.42
Valine	2.96	2.99		2.01	2.02	2.02	2.03	2.04	2.10
^3^ ∑EAA	27.11	26.23		19.50	19.51	19.48	19.44	19.39	19.42
Non-essential amino acid (%)									
Alanine	4.04	4.00		2.88	2.86	2.85	2.83	2.82	2.81
Aspartic acid	5.58	5.00		4.33	4.30	4.27	4.25	4.23	4.22
Cysteine	0.80	0.92	0.12 ^2^	0.69	0.70	0.71	0.73	0.74	0.74
Glutamic acid	8.08	8.03		7.15	7.10	7.08	7.07	7.05	7.02
Glycine	3.94	4.99		2.72	2.84	2.86	2.93	3.10	3.08
Proline	2.65	3.63		2.39	2.33	2.39	2.44	2.45	2.57
Serine	2.46	2.46		2.08	2.10	2.04	2.10	2.14	2.08
Tyrosine	1.53	1.53		1.19	1.19	1.15	1.16	1.19	1.15
^4^ ∑NEAA	29.08	30.56		23.43	23.42	23.35	23.51	23.72	23.67

^1^ Data was obtained from Yan et al.’s [30] study. ^2^ Data were obtained from Yan et al.’s [31] study. ^3^ ∑EAA: Total essential amino acids. ^4^ ∑NEAA: Total non-essential amino acids.

**Table 3:** Fatty acid (FA) profiles (% of total fatty acids) of the main ingredients (FM and CBM) and experimental diets when replacing various levels of fish meal with chicken by-product meal.

	Ingredients		Requirement	Experimental Diets					
	FM	CBM		Con	CBM_10_	CBM_20_	CBM_30_	CBM_40_	CBM_50_
C14:0	5.93	1.78		2.90	2.83	2.61	2.37	2.20	1.99
C16:0	23.07	26.18		17.00	18.00	18.20	18.69	19.07	19.36
C18:0	4.18	8.52		3.81	4.16	4.42	4.74	4.90	5.19
C20:0	0.13	0.16		0.19	0.16	0.13	0.11	0.17	0.23
C22:0	0.19	0.17		0.28	0.27	0.27	0.27	0.27	0.28
C24:0	0.74	0.28		0.61	0.60	0.58	0.54	0.49	0.48
∑SFA ^1^	34.24	37.09		24.79	26.02	26.21	26.72	27.10	27.53
C16:1n-7	6.82	7.27		3.83	4.03	4.17	4.42	4.55	4.62
C18:1n-9	14.38	40.51		25.66	26.83	29.07	31.15	32.72	34.52
C20:1n-9	2.05	0.51		1.54	1.53	1.41	1.34	1.23	1.17
C22:1n-9	0.12	0.34		0.03	0.04	0.04	0.05	0.06	0.06
C24:1n-9	1.61	0.11		0.78	0.73	0.68	0.62	0.58	0.54
∑MUFA ^2^	24.98	48.74		31.84	33.16	35.37	37.58	39.14	40.91
C18:2n-6	2.95	11.28		23.55	21.94	21.26	20.32	19.76	19.02
C18:3n-3	0.87	0.64		3.21	2.91	2.72	2.52	2.38	2.20
C20:4n-6	3.90	1.38		2.19	2.16	2.13	2.02	1.89	1.72
C20:5n-3	12.76	0.04		4.96	4.70	4.18	3.64	3.24	2.80
C22:2n-6	0.65	0.04		0.34	0.33	0.35	0.33	0.30	0.27
C22:6n-3	15.84	0.13		6.96	6.45	5.71	4.99	4.40	3.80
∑n-3 HUFA ^3^	28.60	0.17	7.20 ^4^	11.92	11.15	9.89	8.63	7.64	6.60
Unknown	3.81	0.66		2.16	2.33	2.07	1.88	1.79	1.75

^1^ ∑SFA: Total saturated fatty acids. ^2^ ∑MUFA: Total monounsaturated fatty acids. ^3^ ∑n-3 HUFA: Total n-3 highly unsaturated fatty acids. ^4^ Data was obtained in Lee et al.’s [32] study, in which the dietary ∑n-3 HUFA requirement for rockfish was estimated to be 0.9% of the diet, this being equivalent to 7.20% of total fatty acids in this study.

**Table 4:** Survival (%), feed efficiency ratio (FER), protein efficiency ratio (PER), protein retention (PR), condition factor (*K*), hepatosomatic index (HSI), and viscerosomatic index (VSI) of rockfish fed the experimental diets replacing various levels of fish meal with chicken by-product meal for 56 days.

	Experimental Diets								Orthogonal Polynomial Contrast			Regression Analysis		
	Con	CBM_10_	CBM_20_	CBM_30_	CBM_40_	CBM_50_	SEM	*p*-Value	Linear	Quadratic	Cubic	Model	*p*-Value	Adj. R^2^
Initial weight (g/fish)	2.4	2.3	2.4	2.4	2.4	2.3	0.01	-	-	-	-	-	-	-
Final weight (g/fish)	12.6 ^a^	12.3 ^a^	12.2 ^a^	11.0 ^b^	10.7 ^b^	9.6 ^c^	0.26	<0.001	0.001	0.028	0.596	L	<0.001	0.856
Survival (%)	95.6	96.7	95.6	97.8	94.4	96.7	0.56	>0.7	0.864	0.451	0.490	NR	-	-
FER ^1^	1.04 ^a^	1.04 ^a^	1.04 ^a^	1.01 ^ab^	1.00 ^b^	0. 98 ^b^	0.01	<0.001	0.001	0.025	0.222	L	<0.001	0.657
PER ^2^	2.06 ^ab^	2.06 ^ab^	2.08 ^a^	2.00 ^abc^	2.00 ^bc^	1.96 ^c^	0.01	<0.002	0.001	0.121	0.355	L	<0.001	0.617
PR (%) ^3^	32.3	31.7	32.4	30.6	30.8	29.7	0.39	>0.3	0.121	0.648	0.982	NR	-	-
*K* (g/cm^3^) ^4^	1.55	1.58	1.53	1.56	1.62	1.57	0.01	>0.6	0.426	0.892	0.575	NR	-	-
HSI (%) ^5^	2.18	2.47	2.55	2.59	2.78	2.48	0.06	>0.1	0.127	0.116	0.675	NR	-	-
VSI (%) ^6^	9.44	9.61	9.43	9.65	9.69	9.34	0.09	>0.9	0.973	0.483	0.584	NR	-	-

Values (means of triplicate) in the same row with different superscript letters represent significant differences (*p <* 0.05). Abbreviations: SEM, pooled standard error of treatment means; Adj. R^2^, adjusted R square; L, linear; NR, no relationship. ^1^ Feed efficiency ratio (FER) = Weight gain of rockfish/feed supplied. ^2^ Protein efficiency ratio (PER) = Weight gain of rockfish/protein supplied. ^3^ Protein retention (PR, %) = Protein gain of rockfish ×100/protein supplied. ^4^ Condition factor (*K*, g/cm^3^) = Body weight of rockfish (g) × 100/total length of rockfish (cm)^3^. ^5^ Hepatosomatic index (HSI, %) = Liver weight of rockfish ×100/body weight of rockfish. ^6^ Visceralsomatic index (VSI, %) = Viscera weight of rockfish × 100/body weight of rockfish.

**Table 5:** Proximate composition (% of wet weight) of rockfish fed the experimental diets replacing various levels of fish meal with chicken by-product meal for 56 days.

	Experimental Diets								Orthogonal Polynomial Contrast		
	Con	CBM_10_	CBM_20_	CBM_30_	CBM_40_	CBM_50_	SEM	*p*-Value	Linear	Quadratic	Cubic
Moisture	73.2	72.8	73.1	72.4	72.0	72.8	0.18	>0.4	0.169	0.463	0.295
Crude protein	15.6	15.4	15.5	15.3	15.4	15.2	0.12	>0.9	0.440	0.935	0.757
Crude lipid	6.9	7.1	6.9	6.8	7.2	7.3	0.07	>0.1	0.090	0.215	0.422
Ash	4.3	4.1	4.0	4.0	4.3	4.1	0.12	>0.9	0.841	0.627	0.544

Abbreviations: SEM, pooled standard error of treatment means.

**Table 6:** AA profiles (% of wet weight) of the whole bodies of rockfish fed the experimental diets, replacing various levels of fish meal with chicken by-product meal for 56 days.

	Experimental Diets								Orthogonal Polynomial Contrast		
	Con	CBM_10_	CBM_20_	CBM_30_	CBM_40_	CBM_50_	SEM	*p*-Value	Linear	Quadratic	Cubic
Essential amino acid (%)											
Arginine	0.94	0.93	0.88	0.90	0.92	0.94	0.01	>0.6	0.989	0.117	0.940
Histidine	0.32	0.32	0.31	0.33	0.31	0.33	0.01	>0.9	0.923	0.790	0.952
Isoleucine	0.58	0.55	0.55	0.58	0.53	0.58	0.01	>0.7	0.913	0.451	0.874
Leucine	1.08	1.04	1.04	1.10	1.02	1.08	0.01	>0.6	0.966	0.611	0.798
Lysine	1.20	1.16	1.15	1.23	1.15	1.21	0.02	>0.7	0.824	0.581	0.794
Phenylalanine	0.57	0.59	0.57	0.59	0.56	0.57	0.01	>0.9	0.853	0.783	0.644
Threonine	0.70	0.68	0.67	0.71	0.67	0.69	0.01	>0.8	0.915	0.818	0.662
Tryptophan	0.10	0.09	0.09	0.09	0.09	0.05	0.01	>0.8	0.327	0.610	0.592
Valine	0.67	0.64	0.63	0.66	0.62	0.67	0.02	>0.9	0.951	0.440	0.987
Non-essential amino acid (%)											
Alanine	1.03	1.04	0.99	1.01	1.03	1.02	0.02	>0.9	0.880	0.685	0.987
Aspartic acid	1.47	1.44	1.42	1.49	1.43	1.46	0.01	>0.7	0.869	0.700	0.542
Glutamic acid	2.03	1.96	1.96	2.07	1.95	2.01	0.02	>0.1	0.941	0.633	0.361
Glycine	1.20	1.25	1.11	1.09	1.28	1.19	0.02	>0.09	0.941	0.171	0.834
Proline	0.69	0.70	0.67	0.66	0.72	0.70	0.01	>0.9	0.785	0.583	0.860
Serine	0.72	0.71	0.69	0.72	0.72	0.70	0.01	>0.9	0.907	0.936	0.598
Tyrosine	0.40	0.38	0.38	0.41	0.38	0.41	0.01	>0.9	0.799	0.731	0.826

Abbreviations: SEM, pooled standard error of treatment means.

**Table 7:** FA profiles (% of total fatty acids) of the whole bodies of rockfish fed the experimental diets, replacing various levels of fish meal with chicken by-product meal for 56 days.

	Experimental Diets								Orthogonal Polynomial Contrast			Regression Analysis		
	Con	CBM_10_	CBM_20_	CBM_30_	CBM_40_	CBM_50_	SEM	*p*-Value	Linear	Quadratic	Cubic	Model	*p*-Value	Adj. R^2^
C14:0	2.39 ^a^	2.28 ^a^	2.20 ^ab^	2.07 ^bc^	2.00 ^bc^	1.92 ^c^	0.04	<0.001	0.001	0.680	0.840	L	<0.001	0.864
C16:0	15.84 ^d^	15.92 ^cd^	16.28 ^bc^	16.43 ^ab^	16.56 ^ab^	16.72 ^a^	0.08	<0.001	0.001	0.491	0.564	L	<0.001	0.846
C18:0	4.58 ^d^	4.60 ^cd^	4.76 ^bc^	4.77 ^b^	4.80 ^b^	4.99 ^a^	0.04	<0.001	0.001	0.265	0.222	L	<0.001	0.791
C20:0	0.22 ^a^	0.23 ^a^	0.17 ^ab^	0.15 ^b^	0.13 ^b^	0.12 ^b^	0.01	<0.001	0.001	0.517	0.108	L	<0.001	0.741
C22:0	0.47	0.37	0.45	0.48	0.39	0.38	0.01	>0.05	0.166	0.364	0.090	NR	-	-
C24:0	0.34	0.31	0.33	0.32	0.32	0.34	0.01	>0.8	0.707	0.348	0.774	NR	-	-
∑SFA^1^	23.84 ^b^	23.71 ^b^	24.19 ^ab^	24.23 ^ab^	24.20 ^ab^	24.48 ^a^	0.07	<0.02	0.001	0.995	0.824	L	<0.001	0.498
C16:1n-7	4.52 ^d^	4.57 ^cd^	4.65 ^cd^	4.73 ^bc^	4.90 ^ab^	5.01 ^a^	0.04	<0.001	0.001	0.073	0.829	L	<0.001	0.875
C18:1n-9	29.12 ^f^	32.21 ^e^	32.97 ^d^	35.03 ^c^	36.43 ^b^	37.90 ^a^	0.70	<0.001	0.001	0.001	0.001	L	<0.001	0.975
C20:1n-9	2.84 ^a^	2.64 ^b^	2.57 ^bc^	2.43 ^cd^	2.31 ^de^	2.24 ^e^	0.05	<0.001	0.001	0.149	0.795	L	<0.001	0.927
C22:1n-9	0.25	0.24	0.28	0.29	0.29	0.27	0.01	>0.3	0.109	0.269	0.357	NR	-	-
C24:1n-9	1.03 ^a^	0.92 ^b^	0.91 ^bc^	0.83 ^cd^	0.77 ^de^	0.73 ^e^	0.03	<0.001	0.001	0.506	0.667	L	<0.001	0.909
∑MUFA ^2^	37.76 ^f^	40.58 ^e^	41.39 ^d^	43.31 ^c^	44.70 ^b^	46.16 ^a^	0.67	<0.001	0.001	0.001	0.001	L	<0.001	0.979
C18:2n-6	20.37 ^a^	18.60 ^b^	18.26 ^b^	17.37 ^c^	17.27 ^c^	16.56 ^d^	0.30	<0.001	0.001	0.001	0.004	Q	<0.001	0.930
C18:3n-3	2.50 ^a^	2.31 ^ab^	2.14 ^bc^	1.94 ^cd^	1.90 ^de^	1.68 ^e^	0.07	<0.001	0.001	0.427	0.558	L	<0.001	0.916
C20:4n-6	1.91 ^a^	1.81 ^ab^	1.76 ^b^	1.73 ^b^	1.73 ^b^	1.68 ^b^	0.02	<0.002	0.001	0.097	0.312	L	<0.001	0.662
C20:5n-3	4.38 ^a^	4.20 ^ab^	3.91 ^bc^	3.58 ^cd^	3.25 ^de^	2.84 ^e^	0.13	<0.001	0.001	0.192	0.842	L	<0.001	0.919
C22:2n-6	0.45 ^a^	0.39 ^ab^	0.36 ^ab^	0.34 ^ab^	0.29 ^b^	0.30 ^b^	0.02	<0.03	0.001	0.309	0.968	L	<0.002	0.528
C22:6n-3	6.28 ^a^	6.07 ^ab^	5.81 ^b^	5.09 ^c^	4.67 ^d^	4.04 ^e^	0.20	<0.001	0.001	0.001	0.133	Q	<0.001	0.971
∑n-3 HUFA ^3^	10.66 ^a^	10.28 ^ab^	9.72 ^b^	8.67 ^c^	7.92 ^c^	6.89 ^d^	0.33	<0.001	0.001	0.025	0.436	L	<0.001	0.943
Unknown	2.51	2.32	2.18	2.42	2.00	2.25	0.06	>0.08	0.053	0.306	0.993	NR	-	-

Values (means of triplicate) in the same row with different superscript letters represent significant differences (*p <* 0.05). Abbreviations: SEM, pooled standard error of treatment means; Adj. R^2^, adjusted R square; L, linear; Q, quadratic; NR, no relationship.^1^ ∑SFA: Total saturated fatty acids.^2^ ∑MUFA: Total monounsaturated fatty acids.^3^ ∑n-3 HUFA: Total n-3 highly unsaturated fatty acids.

**Table 8:** Plasma and serum parameters of rockfish fed the experimental diets, replacing various levels of fish meal with chicken by-product meal for 56 days.

	Experimental Diets								Orthogonal Polynomial Contrast		
	Con	CBM_10_	CBM_20_	CBM_30_	CBM_40_	CBM_50_	SEM	*p*-Value	Linear	Quadratic	Cubic
Plasma parameters											
AST (U/L)	150.7	151.3	150.3	151.0	147.7	148.3	0.71	>0.6	0.174	0.586	0.651
ALT (U/L)	24.3	24.0	23.0	23.3	25.3	22.3	0.46	>0.5	0.563	0.900	0.201
ALP (U/L)	184.3	189.7	186.3	189.3	189.3	189.0	0.74	>0.1	0.087	0.334	0.543
TB (mg/dL)	1.8	1.3	1.7	1.5	1.2	1.2	0.14	>0.7	0.260	0.907	0.699
T-CHO (mg/dL)	253.3	256.0	252.7	255.7	253.7	252.7	0.58	>0.4	0.545	0.334	0.959
TG (mg/dL)	363.3	364.0	360.7	361.0	364.7	360.7	0.66	>0.2	0.408	0.746	0.366
TP (g/dL)	4.6	4.2	4.7	4.2	4.6	4.3	0.13	>0.7	0.722	0.999	0.571
ALB (g/dL)	1.2	1.1	1.5	1.1	1.1	1.0	0.08	>0.5	0.557	0.370	0.790
Serum parameters											
SOD (ng/mL)	2.5	2.4	2.7	2.5	2.5	2.6	0.07	>0.8	0.627	0.905	0.639
Lysozyme (U/mL)	399.9	346.2	485.2	304.3	366.6	333.8	26.07	>0.4	0.417	0.735	0.775

Abbreviations: AST, aspartate aminotransferase; ALT, alanine aminotransferase; ALP, alkaline phosphatase; TB, total bilirubin; T-CHO, total cholesterol; TG, triglyceride; TP, total protein; ALB, albumin; and SOD, superoxide dismutase. SEM, pooled standard error of treatment means.

**Table 9:** Effects of dietary treatments on economic parameters of the study.

	Experimental Diets								Orthogonal Polynomial Contrast			Regression Analysis		
	Con	CBM_10_	CBM_20_	CBM_30_	CBM_40_	CBM_50_	SEM	*p*-Value	Linear	Quadratic	Cubic	Model	*p*-Value	Adj. R^2^
Diet price (USD/kg)	1.65	1.58	1.51	1.44	1.37	1.29	-	-	-	-	-	-	-	-
ECR (USD/kg) ^1^	1.57 ^a^	1.50 ^b^	1.43 ^c^	1.42 ^c^	1.34 ^d^	1.30 ^d^	0.11	<0.001	0.001	0.035	0.551	L	<0.001	0.878
EPI (USD/fish) ^2^	1.24 ^a^	1.21 ^a^	1.21 ^a^	1.08 ^b^	1.05 ^b^	0.95 ^c^	0.11	<0.001	0.001	0.030	0.564	L	<0.001	0.852

Values (means of triplicate) in the same row with different superscript letters represent significant differences (*p <* 0.05). Abbreviations: SEM, pooled standard error of treatment means; Adj. R^2^, adjusted R square; L, linear; Q, quadratic. ^1^ Economic conversion ratio (ECR, USD/kg) = Feed consumption (kg/fish) × diet price (USD/kg)/weight gain of fish (kg/fish). ^2^ Economic profit index (EPI, USD/fish) = Final weight (kg/fish) × fish sale price (USD/kg)—feed consumption (kg/fish) × diet price (USD/kg).

## Data Availability

The data that support the findings of this study are available from the corresponding author upon reasonable request.
